# Supplementary material for: Recombinant Forms of HIV-1 in the Last Decade of the Epidemic in the Russian Federation
Source: Viruses. 2023 Nov 25;15(12):2312. doi: 10.3390/v15122312 (PMC10748268; doi:10.3390/v15122312)
Supplement: Supplementary file 1 [file viruses-15-02312-s001.zip › viruses-2707306-supplementary.pdf]

Table S1. PCR conditions for obtaining *pol* gene amplicons.

Coding region	Round	Primers	Amplification modes
PR–RT	1	RP1S/RP1S	95 °C – 5 min<br>94 °C – 30 s<br>55 °C – 1 min<br>72 °C – 1 min 30 s<br>} 35 cycles<br>72 °C – 10 min<br>4 °C – ∞
	2	PROS2/RTOA	95 °C –5 min<br>94 °C – 30 s<br>51 °C – 1 min<br>72 °C – 1 min<br>} 35 cycles<br>72 °C – 5 min<br>4 °C – ∞
PR	1	POM/R2726	95 °C – 2 min<br>95 °C – 30 s<br>56 °C – 1 min<br>72 °C – 1 min<br>} 35 cycles<br>72 °C – 7 min<br>4 °C – ∞
	2	F2111/polR1	95 °C – 2 min<br>95 °C – 30 s<br>55 °C – 1 min<br>72 °C – 1 min 30 s<br>} 35 cycles<br>72 °C – 7 min<br>4 °C – ∞
RT	1	F2491/RT2A	95 °C – 2 min<br>95 °C – 30 s<br>49 °C – 1 min<br>72 °C – 2 min<br>} 35 cycles<br>72 °C – 7 min<br>4 °C – ∞
	2	RT1A/R3271	95 °C – 2 min<br>95 °C – 30 s<br>46 °C – 1 min<br>72 °C – 2 min<br>} 35 cycles<br>72 °C – 7 min<br>4 °C – ∞

Table S2. Reference nucleotide sequences used for recombination analysis (RDP4).

HIV-1 genetic variant	Reference nucleotide sequences (number in the international database of the Los Alamos Laboratory)
A1	A1.KE.1994.Q23_17.AF004885<br>A1.SE.1994.SE7253.AF069670<br>A1.UG.1998.98UG57136.AF484509<br>A1.UG.1992.92UG037.U51190
A6	A6.UA.2000.98UA0116.AF413987<br>A6.GE.1999.99GEMZ011.DQ207944<br>A6.IT.2002.60000.EU861977<br>A6.BY.2013.PV85.KT983615
B	*B pandemic:*<br>B.US.1986.5019_86.AY835780<br>B.US.1983.RF_HAT3.M17451<br>*B-FSU (MSM):*<br>B.UA.2001.01UAKV252.DQ823363<br>B.UA.2001.01UAKV259.DQ823364<br>*B-FSU (IDU):*<br>B.UA.2001.01UAKV259.DQ823364<br>B.RU.2011.11RU21n.JX500708<br>*B Carribean:*<br>B.HT.2005.05HT_129389.EU839602<br>B.JM.2005.05JM_KJ108.EU839605<br>*B Thailand:*<br>B.TH.2005.05TH429730.JN248347<br>B.TH.1996.M041.DQ354114<br>B.TH.2005.05TH429730.JN248347
G	G.KE.1993.HH8793_1_1.AF061640<br>G.BE.1996.DRCBL.AF084936<br>G.NG.1992.92NG083_JV10832.U88826<br>G.SE.1993.SE6165_G6165.AF061642
CRF03_AB	03_A6B.RU.1997.KAL153_2.AF193276
CRF02_AG	02_AG.NG.-.IBNG.L39106
CRF63_02A6	63_02A6.RU.2010.10RU6637.JN230353

Table S3. Characterization of HIV-1 unique recombinant forms identified in the study.

Sequence name in GenBank	Genome fragment (subtype)			The genome map of the *pol* gene region studied
	1	2	3	
AB-unique recombinant forms				
KZN014.KJ466558	2253-2450 (Thai-B)	2451-3551 (A6)	-	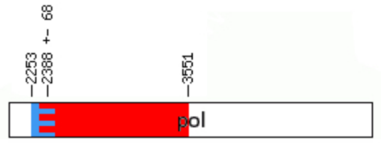
ARH008.KU557644	2253-3233 (A6)	3234-3485 (B-FSU)	-	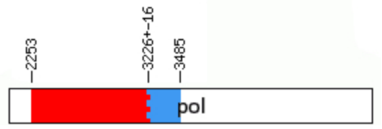
ARH025.KU933330	2253-2992 (Car-B)	2993-3233 (A6)	-	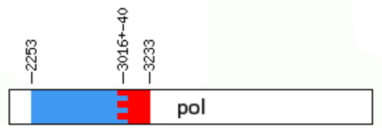
ARH040.KU670323	2253-3226 (CRF03)	3227-3506 (A6)	-	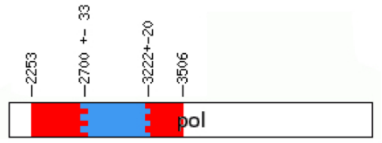
CHR019.MN187975	2253-3255 (CRF03)	3256-3551 (A6)	-	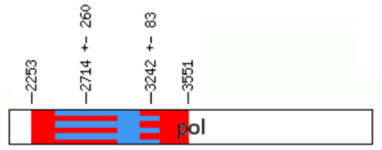
CHR070.MN187978	2253-2913 (A6)	2914-3202 (Thai-B)	3203-3551 (A6)	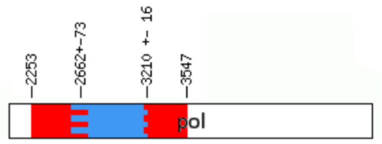
CHR080.MN187980	2253-3199 (CRF03)	3200-3547 (A6)	-	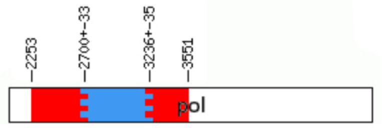
CHR166.MN187986	2253-3255 (CRF03)	3256-3551 (A6)	-	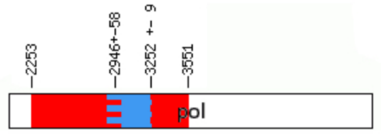
CHR556.MN188005	2253-2952 (A6)	2953-3255 (Thai-B)	3256-3551 (A6)	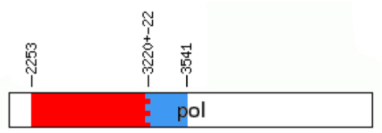
SHL057.MF124853	2253-3221 (A6)	3222-3541 (B pandemic)	-	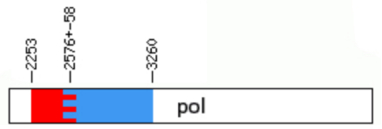
SHL061.MF124856	2253-2546 (A6)	2547-3260 (Car-B)	-	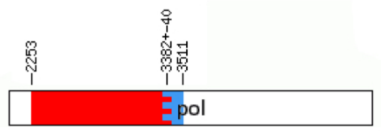

74-2007.OM050092	2253-3386 (A6)	3387-3511 (B-FSU)	-	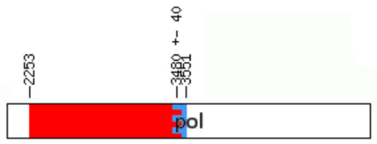
KRS4269.MK589439	2253-3478 (A6)	3479-3551 (B pandemic)	-	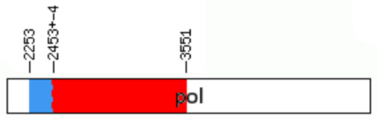
NNG561.KJ722105	2253-2450 (B pandemic)	2451-3551 (A6)	-	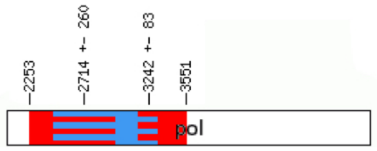
3532.OK474476	2253-2609 (A6)	2610-3171 (B-FSU)	-	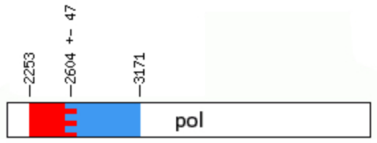
1703A.OK474378	2253-2609 (A6)	2610-3171 (B-FSU)	-	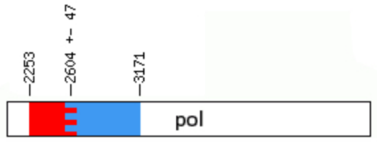
4362A.OK474517	2253-2609 (A6)	2610-3171 (B-FSU)	-	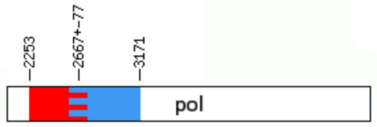
KRD209.MK795075	2253-2556 (A6)	2557-3232 (Car-B)	-	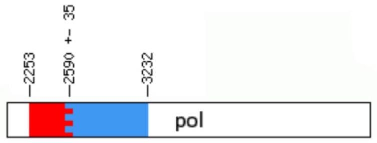
3570D OK474482	2253-2952 (A6)	2953-3171 (B-Thai)	-	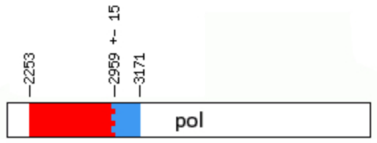
2713A.OK474433	2253-2554 (Thai-B)	2555-3050 (A6)	3051-3171 (Thai-B)	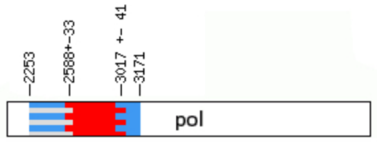
1312000070.OK474725	2253-3030 (A6)	3031-3171 (B pandemic)	-	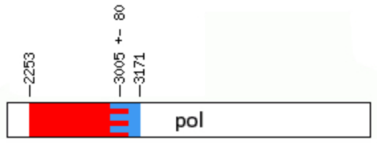
1312000202.OK474750	2253-2556 (A6)	2557-3171 (Car-B)	-	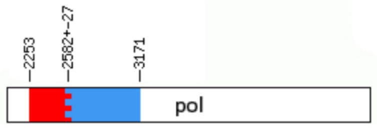
1312000208.OK474753	2253-2556 (A6)	2557-3171 (Car-B)	-	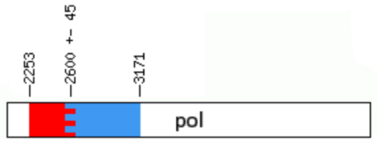

1312000261.OK474757	2253-2654 (A6)	2655-3171 (B-FSU)	-	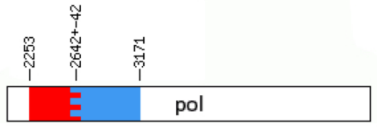
MOW102645.MH666364	2253-2462 (Thai-B)	2463-3551 (A6)	-	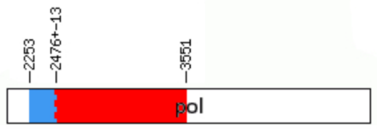
MOW103145.MH666370	2253-2459 (Car-B)	2460-3551 (A6)	-	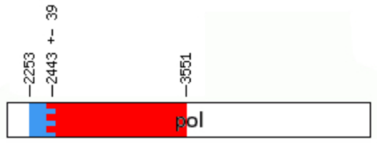
MOW109184.MH666393	2253-2437 (Thai-B)	2438-3551 (A6)	-	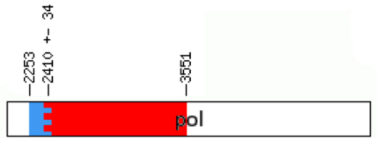
MOW119218.MH666433	2253-3277 (A6)	3278-3551 (B pandemic)	-	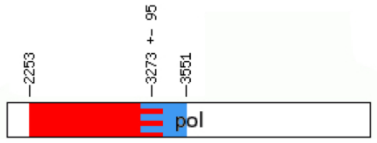
MOW125543.MH666460	2253-2479 (A6)	2480-3046 (Car-B)	3047-3551 (A6)	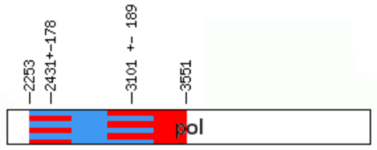
MOW126388.MH666465	2253-3404 (A6)	3405-3551 (B-FSU)	-	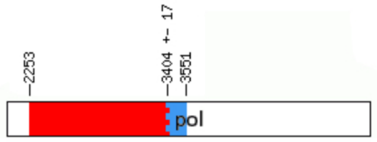
MOW127873.MH666474	2253-3479 (A6)	3480-3551 (B pandemic)	-	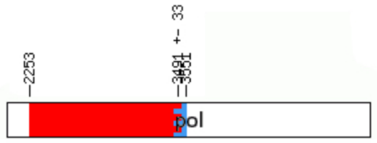
MOW129062.MH666490	2253-3418 (A6)	3419-3551 (B-FSU)	-	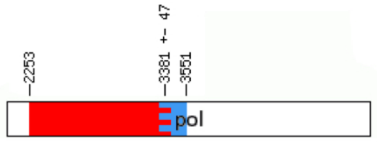
MOW170294.MH666600	2253-2351 (B pandemic)	2352-3551 (A6)	-	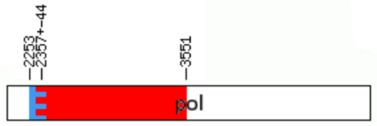
MOW173915.MH666609	2253-2450 (B pandemic)	2451-3551 (A6)	-	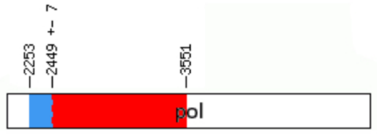
MOW178169.MH666629	2253-3386 (A6)	3387-3551 (B pandemic)	-	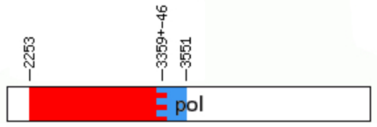

MOW179103.MH666637	2253-3444 (A6)	3445-3551 (B pandemic)	-	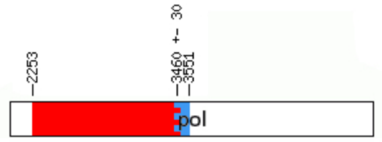
MOW189742.MH666666	2253-3416 (A6)	3417-3551 (B-FSU)	-	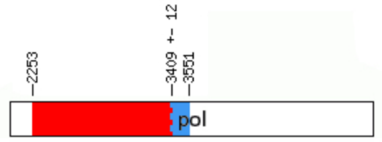
MOW191857.MH666675	2253-3413 (A6)	3414-3551 (B-FSU)	-	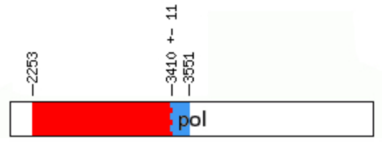
MOW197769.MH666700	2253-3445 (A6)	3446-3551 (Car-B)	-	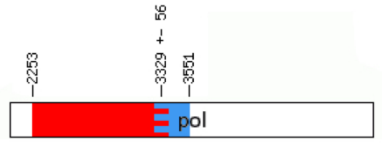
MOW206415.MH666754	2253-3346 (A6)	3347-3551 (B pandemic)	-	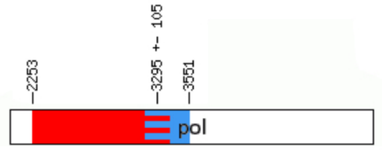
MOW212381.MH666794	2253-2440 (Thai-B)	2441-3551 (A6)	-	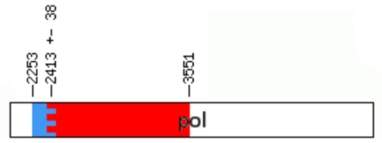
MOW230737.MH666850	2253-3405 (A6)	3406-3551 (B pandemic)	-	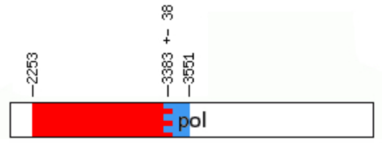
MOW236022.MH666874	2253-3323 (A6)	3324-3551 (B pandemic)	-	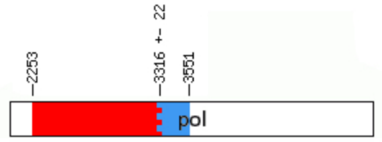
MOW23690.MH666876	2253-3365 (A6)	3366-3551 (B-FSU)	-	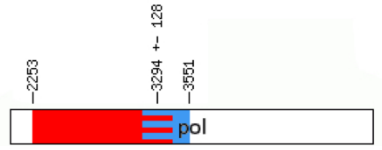
MOW238398.MH666879	2252-3337 (A6)	3338-3551 (Car-B)	-	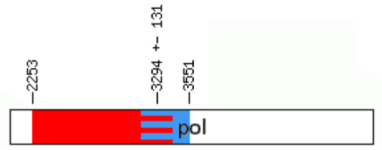
MOW243074.MH666904	2253-3400 (A6)	3401-3551 (B-FSU)	-	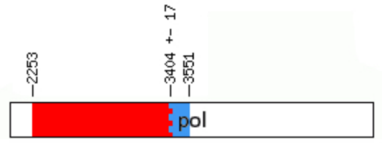
MOW243257.MH666908	2253-3344 (A6)	3345-3551 (B pandemic)	-	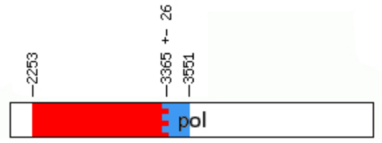

MOW255873.MH666973	2253-2983 (A6)	2984-3203 (B-FSU)	3204-3551 (A6)	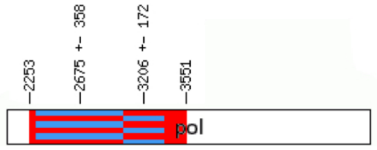
MOW257204.MH666986	2253-2434 (Car-B)	2435-3551 (A6)	-	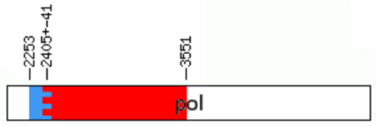
MOW259253.MH667011	2253-3069 (B pandemic)	3070-3212 (A6)	3213-3551 (B pandemic)	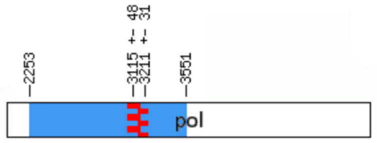
MOW259399.MH667013	2253-3478 (A6)	3479-3551 (B pandemic)	-	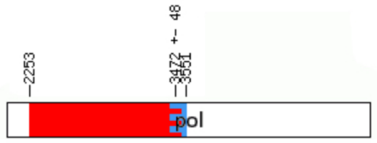
MOW262312.MH667034	2253-2314 (B-FSU)	2315-3551 (A6)	-	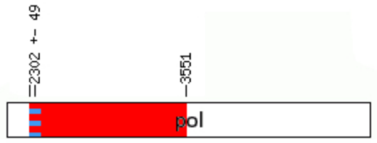
MOW26246.MH667035	2253-2313 (B-FSU)	2314-3551 (A6)	-	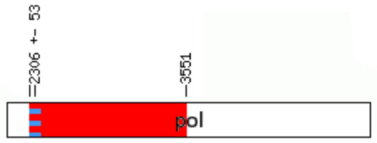
MOW263544.MH667042	2253-2416 (Car-B)	2417-3551 (A6)	-	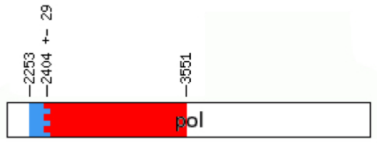
MOW263861.MH667050	2253-2458 (B-FSU)	2459-3551 (A6)	-	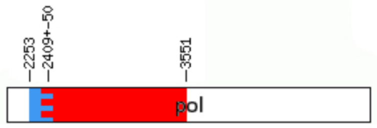
MOW267311.MH667060	2253-3255 (Thai-B)	3256-3551 (A6)	-	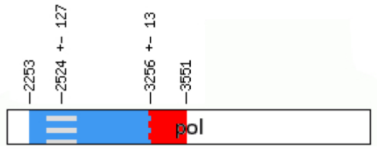
MOW269550.MH667067	2253-2314 (B-FSU)	2315-3551 (A6)	-	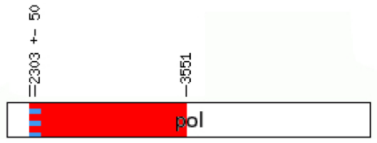
MOW270119.MH667070	2253-2751 (A6)	2752-3085 (B-FSU)	3086-3551 (A6)	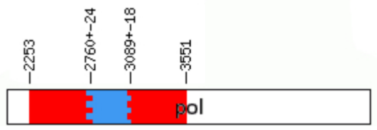
MOW272471.MH667076	2253-2314 (B-FSU)	2315-3551 (A6)	-	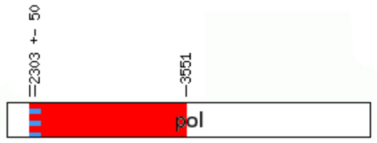

MOW273948.MH667082	2253-3361 (A6)	3362-3551 (B pandemic)	-	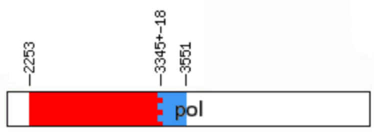
MOW283088.MH667113	2253-2441 (B pandemic)	2442-3551 (A6)	-	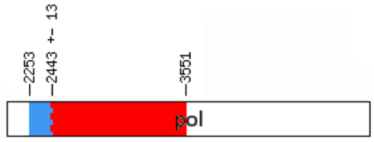
MOW284149.MH667116	2253-3432 (A6)	3433-3551 (Car-B)	-	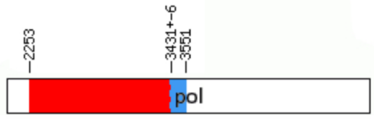
MOW285258.MH667121	2253-2666 (B pandemic)	2667-3551 (A6)	-	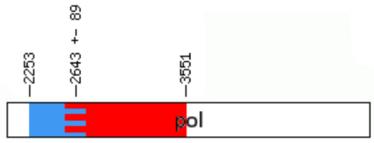
MOW302647.MH667192	2253-2314 (B-FSU)	2315-3551 (A6)	-	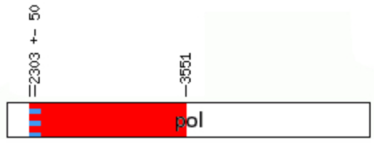
MOW316504.MH667217	2253-2970 (A6)	2971-3267 (Thai-B)	3268-3551 (A6)	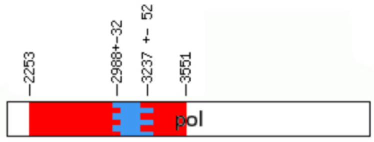
MOW328087.MH667247	2253-3347 (A6)	3348-3551 (B-FSU)	-	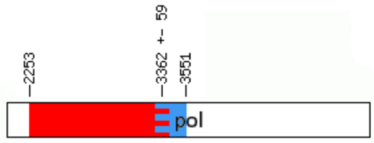
MOW328606.MH667250	2253-3344 (A6)	3345-3551 (B-FSU)	-	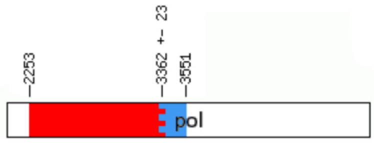
1311000247.MW756410	2253-3267 (Thai-B)	3268-3551 (A6)	-	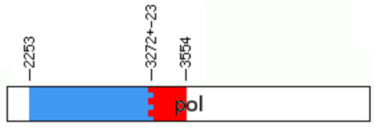
1311000771.OL792445	2253-2963 (A6)	32964-3291 (Thai-B)	3292-3554 (A6)	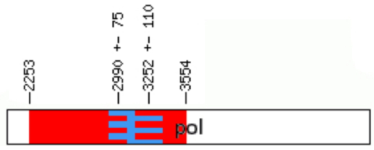
1311001057.OL792566	2253-2556 (A6)	2557-3098 (Car-B)	3099-3554 (A6)	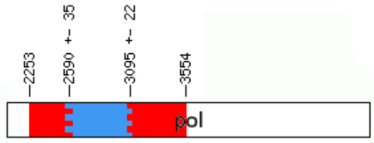

AG-unique recombinant forms				
BRN007.MK612420	2253-2582 (CRF63)	2583-3263 (A6)	-	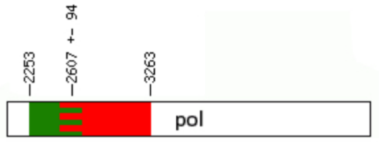
BRN012.MK612425	2253-2519 (CRF63)	2520-3260 (A6)	-	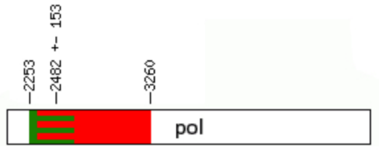
BRN017.MK612430	2253-2893 (CRF63)	2894-3309 (A6)	3310-3611 (CRF63)	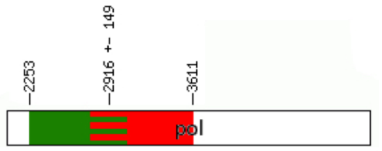
BRN021.MK612434	2253-2954 (A6)	2955-3235 (CRF63)	-	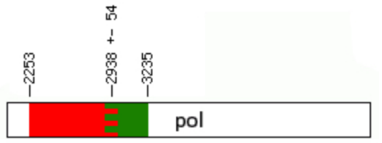
BRN061.MK612464	2253-2545 (CRF63)	2546-3181 (A6)	3182-3263 (CRF63)	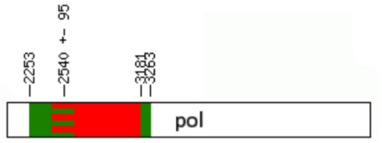
BRN108.MK612479	2253-2392 (CRF63)	2393-3581 (A6)	-	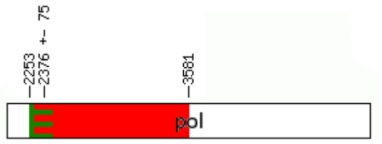
BRN125.MK612484	2253-3179 (G)	3180-3584 (A6)	-	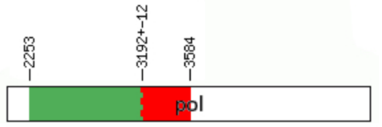
SHL007.MF124826	2253-2624 (A6)	2625-3089 (G)	3090-3545 (A6)	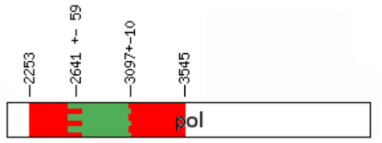
SHL018.MF124832	2253-2512 (A6)	2513-3098 (G)	3099-3545 (A6)	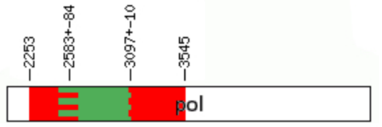
SHL087.MF124872	2253-2841 (A6)	2842-3152 (G)	3153-3545 (A6)	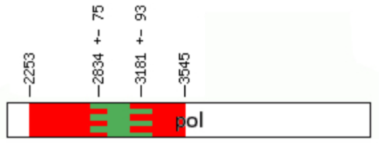
MOW18862.MH666662	2253-2790 (CRF63)	2791-3551 (A6)	-	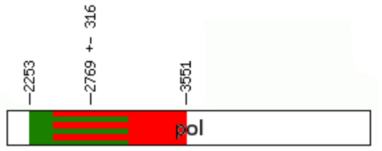
1311001021.OL792536	2253-2386 (CRF63)	2387-2656 (A6)	2657-3554 (CRF63)	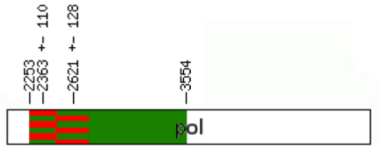

1311001107.OL792603	2253-2357 (CRF63)	2358-2619 (A6)	2620-3554 (CRF63)	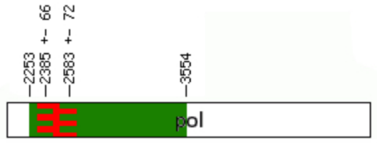
Unique recombinant forms with a mosaic genome structure				
MOW105047.MH666377	2253-2968 (Car-B)	2969-3551 (CRF63)	-	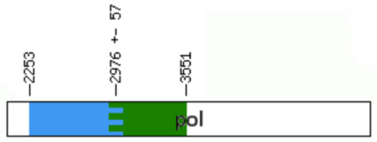
MOW177762.MH666627	2553-3450 (CRF63)	3451-3551 (B pandemic)	-	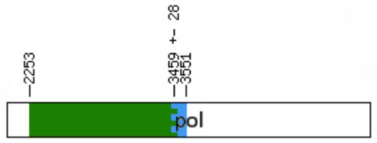
MOW212318.MH666793	2253-2451 (Thai-B)	2452-3551 (CRF02)	-	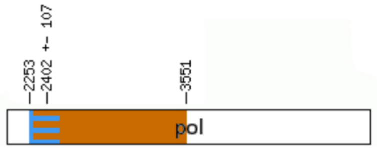
1312000001.OK474695	2253-2876 (CRF02)	2877-3171 (Car-B)	-	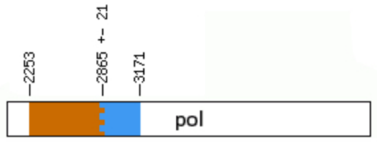

*Sub-subtype A6 is indicated in red, subtype B – in blue, G – in light green color; recombinant form CRF02_AG in brown and CRF63_02A6 in dark green color.

Table S4. Access numbers of all analyzed HIV-1 sequences in the Los Alamos National Laboratory database (GenBank).

City	GenBank Accession number
Arkhangelsk	KU557639-KU557648, KU645849-KU645856, KU645858-KU645883, KU670321-KU670329, KU933329-KU933337, KU933339-KU933340
Barnaul	MK612415-MK612496
Blagoveshchensk	KC254581-KC254615, KC156532-KC156533, KC208003-KC208005
Vladivostok	KC701380-KC701384, KF205368-KF205369, KF205371-KF205374, KF205376-KF205391, KF971919-KF971945, KJ461969-KJ461976
Vologda	MN188025-MN188049, MN188051
Izhevsk	MN235900-MN235941
Irkutsk	KC254616-KC254643
Kazan	KJ499552-KJ499592, KJ499609-KJ499622, KJ499625-KJ499626, KJ499628, KJ499630-KJ499636
Krasnodar	MK795070-MK795125, OK474336-OK474757
Krasnoyarsk	MK589431-MK589711
Moscow region	MH666355-MH666433, MH666435-MH666454, MH666456-MH666510, MH666512-MH666645, MH666647-MH666648, MH666650-MH666683, MH666685-MH666703, MH666705-MH666726, MH666728-MH666768, MH666770-MH666777, MH666779-MH666797, MH666799-MH666809, MH666811-MH666828, MH666830- MH666831, MH666833-MH666842, MH666844-MH666870, MH666872-MH666897, MH666899-MH666901, MH666903-MH666931, MH666933, MH666935-MH666959, MH666961-MH667006, MH667008-MH667086, MH667088-MH667140, MH667142-MH667163, MH667165-MH667169, MH667171, MH667173-MH667200, MH667202-MH667223, MH667225-MH667238, MH667240-MH667241, MH667243-MH667255, KY857892-KY587904, KY857906-KY587907, KY857909-KY587913, KY857915, KY857918-KY587922, MW756383-MW756390, MW756393-MW756414, MW756416-MW756419, MW756421-MW756427, OL792300-OL792570, OL792574-OL792612
Murmansk	KX432074-KX432139
Nizhnevartovsk	MW115451-MW115458, MW115460-MW115466, MW115468-MW115482
Nizhny Novgorod	KJ722070-KJ722139
Noyabrsk	OM049964-OM049965, OM049967, OM049969, OM049972-OM049973, OM049976-OM049979, OM049982-OM049984, OM049988-OM049990, OM049992-OM049997, OM050001-OM050006, OM050008-OM050009, OM050012, OM050014-OM050017, OM050019-OM050022, OM050025-OM050027, OM050031-OM050033, OM050035-OM050046, OM050049-OM050053, OM050056, OM050058-OM050061, OM050063-OM050064, OM050066, OM050068-OM050079, OM050081-OM050092, OM050094-OM050101, OM050103, OM050106-OM050111, OM050113-OM050115, OM050118-OM050121, KX517440-KX517441, KX517443, KX530764, KX530766, MT461102, MT461107
Perm	KP659664-KP659684, KP659686-KP659736, KP659663, KP659685
Simferopol	MK606541, MK606543-MK606563, MK606565-MK606573, MK606575-MK606580, MK606583-MK606586, MK606588-MK606593, MK606595-MK606624
Surgut	MW115483-MW115527
Khabarovsk	KC509837-KC509885, KC665916-KC665944, KF177159-KF177168
Khanty-Mansiysk	MW115433-MN115450
Cherepovets	MN187975-MN188024
Yuzhno-Sakhalinsk	MF124822-MF124874
